# Supplementary material for: Glut-3 Gene Knockdown as a Potential Strategy to Overcome Glioblastoma Radioresistance
Source: Int J Mol Sci. 2024 Feb 8;25(4):2079. doi: 10.3390/ijms25042079 (PMC10889562; doi:10.3390/ijms25042079)
Supplement: Supplementary file 1 [file ijms-25-02079-s001.zip › ijms-2855176-supplementary.pdf]

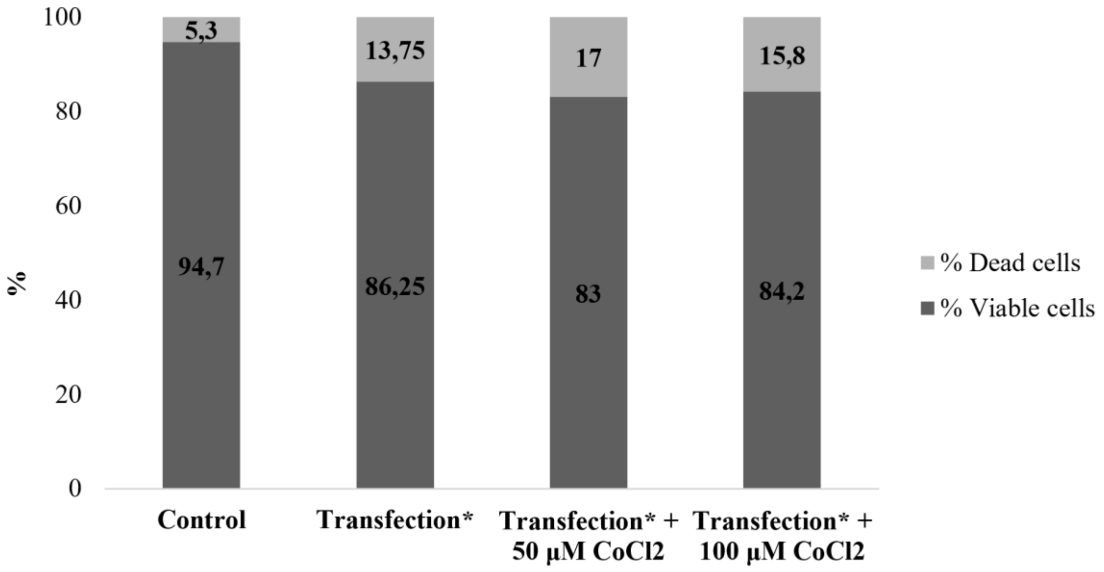

Supplementary file S1. Percentages of viable and dead cells in the control, after the transfection treatment *(1 µl/ml of transfecting agent and 0.8 µg/ml of plasmid DNA) or the co-treatment with transfection and 50 or 100 µM $CoCl_2$.

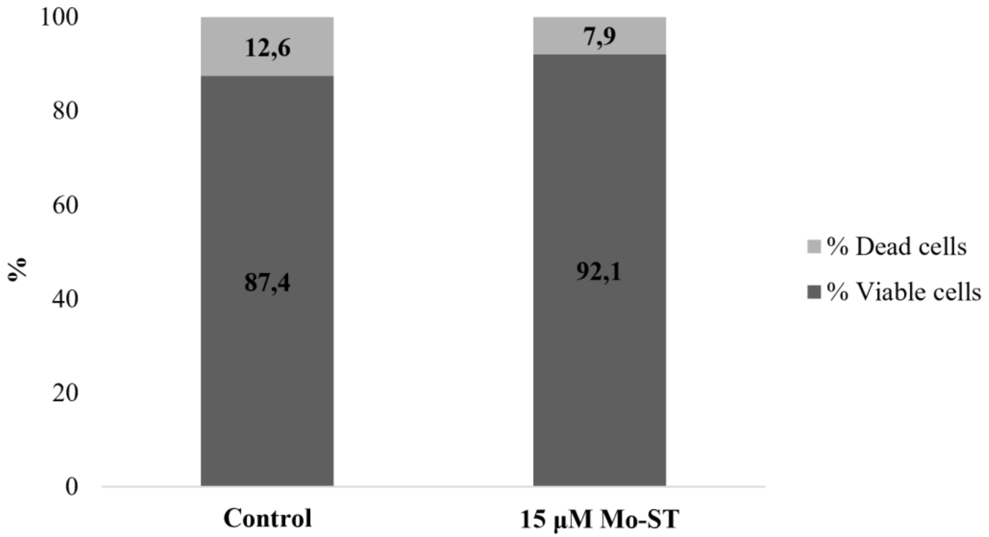

Supplementary file S2. Percentages of viable and dead cells in the control or after the treatment with 15 µM Mo-ST.
